# Seroepidemiological Study on SARS-CoV IgG Antibodies of Different Populations from Several Areas

**Published:** 2005-06

**Authors:** Yu-ling Shi, Lin-hai Li, Xin-wei Chu, Qing Chen, Yun-long Wang, Qing-jun Ma, Cheng Cao, Shou-yi Yu

**Affiliations:** 1 *Clinical Laboratory, Guangzhou General Hospital of PLA, Guangzhou, China;*; 2 *Department of Epidemiology, First Military Medical University, Guangzhou, China;*; 3 *Center of Biotechnology and Engineering of Henan Province, Zhengzhou, China;*; 4 *Institute Biotechnology, Academy of Military Medical Sciences, Beijing, China*

**Keywords:** severe acute respiratory syndrome, serum antibodies, ELISA, IFA

## Abstract

**Objective:**

In order to investigate the clinical and epidemiological rules of severe acute respiratory syndrome (SARS), rates and levels SARS coronavirus (SARS-CoV) IgG antibodies of the patients and community populations from several areas were detected.

**Methods:**

Indirect immunofluorescent assay (IFA) and double-antigen sandwich enzyme-linked immunosorbent assay (ELISA) were used to detect the SARS coronavirus-specific IgG antibodies in sera of 1700, including 1453 general populations from Hongkong, Marco, Guangzhou and Peking and 257 SARS patients from Guangzhou and Peking. The dynamics of the serum antibodies of SARS patients were observed from 3 to 360 days after onset of symptoms.

**Results:**

90% of 257 patient serum specimens after 20 days of disease onset showed positive SARS-CoV IgG either using ELISA or IFA. 257 SARS patients, antibodies titers increased steadily in early 4 to 6 months after onset of SARS. The titers of most cases came to the peak in the 6th month. then antibodies titers declined rapidly in some cases. However, all specimens still were positive for SARS-CoV IgG in the 48th month.

**Conclusions:**

This study suggest that few inapparent infectious patients exist during SARS epidemic. Serum IgG antibodies has diagnostic value for SARS in the late course of disease and the antibodies present more than 48 months.

## INTRODUCTION

Severe Acute Respiratory syndrome (SARS) is a newly discoverable acute respiratory infection, which is also called infectious atypical Pneumonia (IAP) in domestic. The pathogenicity is a new coronavirus, different from any virus of its virus family in the bodies of human beings or animals, namely SARS-CoV (1). Till now, it is not clear about the infecting chart and prevailing rules of SARS-CoV. In order to find out changing rules of the clinical SARS cases serum specific IgG antibodies and whether there is inapparent infection in health population, this study adopts the indirect immunofluorescent assay diagnosis reagent developed by Institute microbe epidemic, Academy of Military Iatrology Sciences (2) and the antigen -capturing enzyme-linked immunosorbent assay by Bioindustry Research Institute, Academy of Military Iatrology Sciences. The results are as follows.

## MATERIALS AND METHODS

### Samples and Sources

The 257 SARS serum samples belonged to infectors hospitalized respectively in General Hospital of Guangzhou Military Region, the 2nd Hospital of Zhongsan University, 177th Hospital of Guangdong Province, the 8th People’s Hospital of Guangzhou Municipal and Peking Tiny Tang Mount Hospital during Feb. and Mar. of 2003. As of the 1453 healthy samples, 935 were from servicemen quartered in Hongkong and 158 from those quartered in Macao in Jun. 2003, 160 were from medical practitioners around Peking that having close contact with SARS infectors and 200 were healthy body checked persons at Guangzhou.

### Method

Serum samples were examined by method of indirect IFA and ELISA (double-antigen sandwich described as SARS-CoV nucleocaspid antigen in Gene-industry). Method of indirect IFA: Dilute the prepared serum with 1:10, extinct under 56°C for 30 minutes, dilute with 1:20, draw 20 ul out, drop in different holes in the antigen piece, set negative and positive comparative serums, warm culture in a 37°C for 30 min, PBS shake and wash 3 times, mixed with isothiocyanic acid Luciferin, warm culture in a 37°C for 30 min, PBS wash 3 times, observe with fluorescence microscope. The antigen -capturing enzyme-linked immunosorbent assay (3) was established for the detection of anti-N protein antibody present in sera. A 100-μl volume of serum was added to the well coated with recombinant N protein, and the plate was incubated at 37°C for 30 min and then washed five times with phosphate-buffered saline containing 0.05% Tween 20. A 10-μl volume of labeled antigen was added,and the plate was incubated for another 30 min and washed as already described, and then 100-μl of TMB substrate solution (0.1mg of tetramethybenzidine hydrochloride/ml, 0.01% H_2_O_2_ in 0.1 M acetate buffer, pH5.8) was added,the mixture was incubated at 37°C for 20 min, the reaction was terminated by adding 50 μl of 2 N sulfuric acid, and the absorbance at 450 nm (A_450_) was determined. Samples with an A_450_ of >0.15 (average A_450_+5 standard deviations of 900 samples from healthy people) were considered to be positive.

## RESULTS

### The positive rate of serum SARS antibodies in healthy people

We examined serums of healthy people with method of double-antigen sandwich ELISA, and found one positive respectively in the 935 servicemen of Hong Kong garrison and in the 158 servicemen of the Macao garrison. But when we tested the two positive samples with method of IFA, we found that they were negative. We detected 160 Health care workers in Peking with ELISA to find that they were all negative. And we detected 200 healthy people in Guangzhou area with both methods of ELISA and IFA, and the results were all negative. Details of the result were listed in Table 1.

**Table 1:** Serum SARS-CoV IgG antibodies test of the health populations using ELISA and IFA

Populations	No.	ELISA		No.	IFA	
		positive positive	%		positive positive	%

Military population in HongKong	935	1	0.1	1	0	0
Military population in Macao	158	1	0.6	1	0	0
Health care workers in Peking	160	0	0	0	-	-
Healthy examination population in Guangzhou	200	0	0	200	0	0

### The positive rate of serum SARS antibodies in patients

We Traced for patients’ serum antibodies using both ELISA and IFA to found that In the first ten days of the disease, 17 serum samples of the 43 cases got positive antibodies, the positive rate was 39.8%. 15 cases were positive by IFA. The positive rate was 34.8%; Within the 11 to 20 days of the disease, 37 samples detected by ELISA and IFA were positive. And the rate was 89%; 20 days later, the positive rates were separately 90.4% and 90.0% by ELASA and IFA in 177 samples. In the early period of the disease, the positive rate detected by IFA was slightly lower than that by ELISA, but the results got close after 10 days of disease. This result showed that it was more meaningful to detect serum IgG antibodies after 11 days, and 89-90% clinical diagnosis patients’ SARS antibodies were positive. The result was detailed in Table 2.

**Table 2:** Serum SARS-CoV IgG antibodies of SARS patients by ELISA and IFA in different time of the course of disease

After onset of disease(days)	No.	ELISA		IFA	
		No. of positive	%	No. of positive	%

3~10	43	17	39.8	15	34.8
11~20	37	33	89.0	33	89.0
21~260	177	160	90.4	159	90.0
22~720	14	14	100	14	100

### Patients’ serum results detected by ELISA

**Figure 1:**
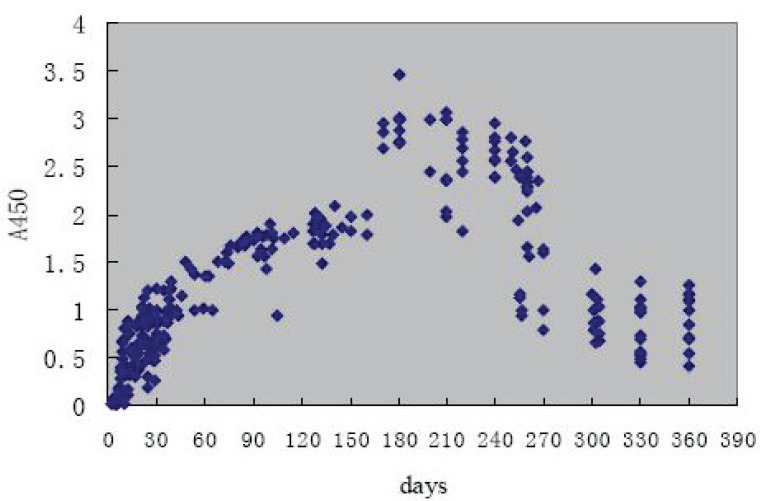
The dynamics of serum SARS-CoV IgG of 257 patients.

**Figure 2:**
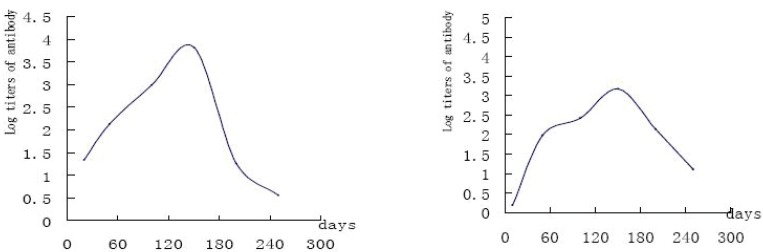
Development of serum SARS CoV-IgG in two SARS patients.

There were 257 SARS cases diagnosed by ELISA. We followed 43 cases of them (after onset of disease, 3-360 days), the result showed that serum IgG antibodies titer was lifted with time went on in the first 4 to 6 months, most of the patients’ antibodies lift to the top at the 6th month. Most of them reached the peak in the 6th month, and a part of them began to drop down but still keep the positive two years (Figure 1 and Figure 2).

## DISCUSSIONS

So far, we know nothing about how SARS came to be and what its infectant and epidemiological rules are. Seroepidemiological Study is the important method to study infectious disease and the prevalence rules. In order to cognize roundly the rules of Serum IgG antibodies and infection charts after onset of SARS, Indirect immunofluorescent assay (IFA) and double-antigen sandwich enzyme-linked immunosorbent assay (ELISA) were used to detect in sera of 1710, including 1453 healthy populations and 257 SARS patients from Guangzhou and Peking.

ELISA method was used to detect the serum samples of 1453 healthy population, including 160 that closely contacted with SARS patients, and only 0.14% of them showed positive SARS-CoV IgG. Due to the false positive result of ELISA, we validated further two positive SARS-CoV IgG serums of them by IFA only to find that the result was negative. We didn't find positive antibodies in 200 serums from Guangzhou district using both IFA and ELISA. This made clear that not the recessive infectious of SARS in the health people.

This study result showed that, of the clinical SARS infected patients in Feb. and Mar. of 2003, at least 90% could be confirmed by serology method. However, we may miss many cases if we detect SARS-CoV IgG antibodies by serology method at the early stage of the disease (first 10 days). Ten days later, serums antibodies appeared in most patients and the results by IFA and ELISA were identical. From the study, we got to the conclusion that the serology method, especially the IFA, has great diagnostic value for the dubious patients diagnosed in clinic and epidemiology (4). Therefore, double serums detection was obviously necessary in SARS lab diagnosis.

By detecting 257 SARS patients from 3 to 360 days after onset of SARS, we found that their serum antibodies titers increased steadily in 4 to 6 months (5). The antibodies of most patients reached the peak in the 6th month and lasted to the 24th month. The antibodies of some patients began to descend after reaching the peak. Though the antibody could be detected positive on the 360th day, the titer declined. This study put forward that the duration of SARS-CoV IgG would last for more than 2 year. We will further trace the falling curve of SARS-CoV IgG antibodies.
